# Interpersonal coordination in schizophrenia: a concise update on paradigms, computations, and neuroimaging findings

**DOI:** 10.1093/psyrad/kkad002

**Published:** 2023-02-07

**Authors:** Yafeng Pan, Yalan Wen, Yajie Wang, Leonhard Schilbach, Ji Chen

**Affiliations:** Department of Psychology and Behavioral Sciences, Zhejiang University, Hangzhou, Zhejiang 310058, China; Department of Psychology and Behavioral Sciences, Zhejiang University, Hangzhou, Zhejiang 310058, China; Department of Psychology and Behavioral Sciences, Zhejiang University, Hangzhou, Zhejiang 310058, China; Department of General Psychiatry 2 and Neuroimaging Section, LVR-Klinikum Düsseldorf, Düsseldorf 40629, Germany; Medical Faculty, Ludwig-Maximilians University, Munich 80539, Germany; Department of Psychology and Behavioral Sciences, Zhejiang University, Hangzhou, Zhejiang 310058, China; Department of Psychiatry, The Fourth Affiliated Hospital, Zhejiang University School of Medicine, Yiwu, Zhejiang 322000, China

**Keywords:** schizophrenia, social functioning, computational psychiatry, neuroimage, interpersonal coordination

Social function deficits are a ubiquitous manifestation of many psychiatric disorders including schizophrenia (Burns, 2006; Green *et al*., 2015; Schilbach, 2016). Patients with schizophrenia (PSZ) exhibit a variety of abnormalities in social cognition related to social perception, facial emotion recognition, mentalization, and interpersonal coordination (Turetsky *et al*., 2007; Schilbach, 2016; Green *et al*., 2019). Aberrant social processing can present challenges in interpersonal interactions for PSZ, leading to poor social adjustment and quality of life (Couture, 2006).

Herein, the term “interpersonal coordination” refers to any actions that occur in spatial and temporal concordance with those of other individuals in a social setting (Ramenzoni *et al*., 2012); as an example, when two people are walking together, they tend to (unintentionally) coordinate their movements. Interpersonal coordination (at least) comprises two interrelated aspects: behavioral matching [i.e. mimicry (e.g. facial expression in response to an emotional stimulus) and imitation (finger tapping along with a human hand)] and interpersonal synchrony (i.e. temporal dynamics of joint actions) (Dean *et al*., 2021). Previous investigations on behavioral matching in schizophrenia have reported conflicting findings; however, they have consistently demonstrated impaired interpersonal synchrony in PSZ (Dean *et al*., 2021). A core feature of interpersonal synchrony is self-organizing temporal dynamism (e.g. the coordination of moment-to-moment behavior with that of another person over time during an interaction); a deficiency in this feature is thought to be a key component of social dysfunction in PSZ. Here, we provide a brief overview of the current state of research on interpersonal coordination in schizophrenia.

## Large-Scale Neuroimaging Investigations for Interpersonal and Social Deficits in the General Population and PSZ

We first point to previous neuroimaging investigations into social deficits in PSZ that have provided solid neural level correlations of this important function. Future studies may leverage *in vivo* brain imaging techniques to further dissect the neurobiological mechanisms underlying particular social interaction processes given recent advances in behavioral paradigms and methodological development.

Whole-brain functional magnetic resonance imaging has been widely used to investigate brain structures and neural circuits involved in psychiatric disorders (Canario *et al*., 2021; Li *et al*., 2021; Chen *et al*., 2022), as well as the neurobiological correlates of social and interpersonal interactions. Systems-level insight into social factors and perceived social isolation in the general populations has been obtained by examining the United Kingdom Biobank imaging genetics cohort (*N* = ∼40 000). A study of the neural signatures of loneliness in this cohort revealed that the default mode network (DMN) is involved in the perception of social isolation: lonely individuals exhibited enhanced functional connectivity in this network and greater integrity of the fornix pathway white matter tract (Spreng *et al*., 2020). A study of social participation as reflected by membership in a social group confirmed that the DMN—including brain regions such as the ventromedial prefrontal cortex, fusiform gyrus, and anterior cingulate cortex along with the limbic network—is important for social belonging (Kieckhaefer *et al*., 2021). It was reported that variations in gray matter volume in brain regions associated with social information processing including the temporoparietal junction, ventro-medial prefrontal cortex, and superior temporal sulcus in middle-aged adults were mainly influenced by socio-environmental factors and activities such as household size and daily routines (Kiesow *et al*., 2021b). The nucleus accumbens, medial prefrontal cortex, and temporoparietal junction were found to be the core regions of stratified social brain subnetworks identified by deep learning that predicted social traits in the general population (Kiesow *et al*., 2021a). An investigation of the trust-based mechanism underlying impaired social interactions in perceived social isolation demonstrated that lonely individuals had reduced limbic and striatal activation and compromised functional connectivity between the anterior insula and occipitoparietal regions during a trust decision task, with a corresponding reduction in affective responses to positive social interactions (Lieberz *et al*., 2021).

In PSZ, decreased connectivity in the DMN comprising large sections of the rostromedial and dorsomedial prefrontal cortex was shown to be associated with social impairment (Saris *et al*., 2022). Using network connectome-based predictive modeling, we previously described social-affective and theory-of-mind networks that reliably predicted symptoms in patients with disorganized schizophrenia (Chen *et al*., 2021). Although the neural basis for interpersonal coordination deficits in schizophrenia remains unclear, the identification of core social brain networks in normal individuals can provide insight into the neural circuits that are dysfunctional in PSZ. One way to bridge the gap between studying the behavioral correlates of social interaction and the neural mechanisms would be by combining functional near-infrared spectroscopy (fNIRS) hyperscanning and behavioral coding (Pan *et al*., 2018; Pan *et al*., 2022a). Fusing brain data and behavioral labels would largely aid the understanding of brain–behavior association in social coordination in PSZ (Pan *et al*., 2022b).

## Recent Methodological Developments in Psychiatry and Interpersonal Coordination

Impairments in social cognition have been investigated using the single-person approach (Green *et al*., 2015): for example, by studying brain activity associated with social ability in an individual (i.e. in a single-person ecologically deprived setting). However, the neurobiological basis of social behaviors cannot be explored in a social vacuum as the brain responds differently to virtual vs. actual stimuli: i.e. their perception in different contexts can elicit distinct types of brain activity (Rolison *et al*., 2020). Understanding the functioning of the social brain requires a comprehensive investigation of the neural features of at least two or multiple individuals during interactions (Kingsbury *et al*., 2019); this so-called “second-person neuroscience” (Schilbach *et al*., 2013) or “interpersonal neuroscience” (Pan *et al*., 2022b) is a promising trend in psychiatry (Schilbach, 2016; Pan & Cheng, 2020; Dumas, 2022).

With the rapid progress in computational neuroscience and interpersonal psychiatry over the past few years (Kriegeskorte & Douglas, 2018; Pan & Cheng, 2020; Dumas, 2022), research on social coordination deficits in PSZ has undergone a methodological evolution from single-person to inter-personal paradigms, and from model-free to model-based analyses. A variety of methods have been used to explore interpersonal coordination in neurotypical populations such as video recordings of interpersonal interactions, tracking of motor movements, psycho-physiological measurements (e.g. breathing, heart rate, and skin response), and neuroimaging (Cornejo *et al*., 2017; Hale *et al*., 2020; Lahnakoski *et al*., 2020; Pan and Cheng, 2020). Using fNIRS hyperscanning to simultaneously evaluate multiple brains, Cui *et al*. (2012) measured interbrain synchrony (i.e. neural processes in one brain that are temporally coupled to neural processes in another brain, Hasson *et al*., 2012; Pan *et al*., 2022b) between two individuals acting coordinately in a button-pressing task. The study of interbrain synchrony can provide rich information about the dynamic interaction and inter-dependencies of two (or more) brains, thus possibly aiding our understanding of impaired inter-personal interaction in PSZ. The finding that interbrain synchrony underlies coordinate behaviors has been replicated and extended in later studies (Cheng *et al*., 2015; Pan *et al*., 2017). However, applying interpersonal neuroscience methods (e.g. finger tapping with fNIRS hyperscanning) to social coordination in clinical patients can be challenging. One difficulty comes from episodic clinical symptoms, which impede the operation of experiment and high-quality data acquisition. By addressing methodological barriers, deciphering true social interaction with translational hyperscanning in PSZ would be possible. A recent fNIRS hyperscanning study has successfully applied interpersonal neuroscience approaches to study social coordination in participants with clinical high risk of psychosis. Results showed reduced interbrain synchrony in the right inferior frontal gyrus when clinical high risk of psychosis coordinated with a healthy control; such reduced interbrain synchrony was associated with symptoms score of suspiciousness/persecutory ideas and movement disorders (Wei *et al*., 2023).

Besides behavioral and neurophysiologic tools, computational models based on mathematical algorithms have provided insight into the neural mechanisms of dynamic interpersonal synchronization. A study using a modified version of the Kuramoto model to analyze self–other integration during interpersonal coordination showed that interpersonal synchronization strategies such as mutual adaptation, leading–following, and leading–leading are associated with different coupling strengths of intrinsic and external action perception processes (Heggli *et al*., 2019). In a follow-up study, the same investigators established a metastable attractor model comprising two predictive submodels—namely, self and other to illustrate the action and perception processes, respectively—to describe the manner in which synchronization strategies of dyads change over time during interactions (Heggli *et al*., 2021). Other studies have used linear models to illustrate complex dynamic interpersonal coordination: one used a context matrix to explain synchrony vs complementation (Miao *et al*., 2022), and the other demonstrated that a balance between synchrony and segregation was important for interpersonal coordination (Mayo and Gordon, 2020).

## Early Findings on Interpersonal Coordination Deficits in Schizophrenia

Beyond findings in neurotypical populations, studies on interpersonal coordination in PSZ have provided insights that are applicable to psychiatric disorders involving atypical social interaction. Interpersonal synchrony depends on multiple complex processes [e.g. perception, simulation, cognition, anticipation, prediction, adaption, mentalization, and execution (Konvalinka *et al*., 2010; Koban *et al*., 2019)], some of which are known to be abnormal in PSZ. For example, a PSZ tapping out a rhythm along with a partner represents a complex interaction pattern, with patients experiencing deficits in sensory input, internal processing, and generation of behaviors. Consequently, PSZ acting in coordination with others may develop atypical synchronization strategies and psychiatric disorders, with impediments in self–other distinction.

Interpersonal coordination deficits have implications for PSZ symptomatology and have been linked to certain dimensions of psychopathology in schizophrenia (Dean *et al*., 2021). Social functions in schizophrenia are heterogeneous and can range from near-normal to severely impaired. Similarly, the degree of negative or positive symptoms in interpersonal coordination tasks can vary across individuals. Negative symptoms are associated with impairment of imitation activities such as gesture performance (Matthews *et al*., 2013), facial expressivity (Falkenberg *et al*., 2008), and accuracy of imitation (Park *et al*., 2007) as well as interactional synchrony, whereby patients are often unable to synchronize their behavior to that of a partner (Cohen *et al*., 2017; Galbusera *et al*., 2018).

Synchrony deficits in PSZ can be investigated using paradigms for interpersonal coordination, such as a simple joint finger-tapping paradigm (Heggli *et al*., 2019) as well as more naturalistic situations (Pan and Cheng, 2020; Lahnakoski *et al*., 2022). However, conventional analytic approaches assess coordination between individuals based solely on reaction time, phase, or synchrony. These parameters are useful but do not reveal the underlying mechanisms of social deficits in schizophrenia—for example, it remains unclear whether social deficits in PSZ are attributable to abnormalities in processing or in the flow of information to and from the healthy partner. Recent methodologic advances in computational modeling provide opportunities to distinguish between these intra- and interpersonal mechanisms of coordination (for details, please refer to Heggli *et al*., 2019).

Early work (before 2021) on interpersonal coordination (especially interactional synchrony) in schizophrenia and related conditions has been reviewed elsewhere (Dean *et al*., 2021), and is only briefly summarized here. Interpersonal coordination has been shown to be impaired in PSZ. In a mirror game with iCub Robot, the occurrence of schizophrenia was reported to be predicted based solely on features of synchronized motor activity with iCub Robot and a computer avatar, with an accuracy and specificity of 93 and 100%, respectively (Słowiński *et al*., 2017). PSZ also showed lower overall synchrony of arm movements with iCub Robot, which was associated with negative symptoms (Cohen *et al*., 2017). This association was confirmed in another study using motion energy analysis, which revealed that decreased synchrony in PSZ was associated with symptom severity and reduced social cognition and functioning and self-esteem (Kupper *et al*., 2015). In an observational study, schizophrenia dyads were unable to synchronize leg movements (Hardin, 1980); however, the abnormal interpersonal synchrony in PSZ was alleviated by implicit exposure to a prosocial primer in a coordination task (Raffard *et al*., 2015). Additionally, prosocial priming enhanced in synchronization partners (age-matched healthy controls) the feeling of connectedness with PSZ. Interactional synchrony, as determined by motion energy analysis, was also improved by body-oriented psychotherapy for schizophrenia (Galbusera *et al*., 2018).

## Challenges and Future Directions

In the last few years, interpersonal tasks, large-scale neuroimaging, and computational models have been used to investigate the mechanisms of social and interpersonal functions in healthy populations. Such multi-modal integration has been noticed and highlighted in our previous perspective (Schilbach, 2016). However, a synthesis of these approaches in empirical studies on clinical psychiatric cohorts remains to be conducted, despite existing studies on social functioning in PSZ (Schwarz *et al*., 2020; Durand *et al*., 2021; Hu *et al*., 2022). Computational methods in schizophrenia have mostly been generative models based on the Bayesian predictive coding framework—for example, the hierarchical Gaussian filtering approach (Powers *et al*., 2017; Henco *et al*., 2020; Charlton *et al*., 2022; Sheffield *et al*., 2022), reinforcement learning (Pratt *et al*., 2021; Geana *et al*., 2022), and the active inference Markov decision process model that attempts to dissect unobservable mechanistic variables based on actions taken by an agent to promote desired outcomes (Friston *et al*., 2016). All of these approaches use a single-person task design and mainly target hallucinatory and delusional positive symptoms, reward-related decision-making, and thought and language deficits in PSZ (Siemerkus *et al*., 2019; Deserno *et al*., 2020; Smith *et al*., 2021; Charlton *et al*., 2022; Knolle *et al*., 2022; Limongi *et al*., 2022). A recent study examining guilt-related interpersonal dysfunction in obsessive-compulsive personality disorder using social interaction tasks applied two computational models (guilt aversion and Fehr–Schmidt inequity aversion models), and demonstrated that interpersonal dysfunction was the result of maladjustment to and poor compliance with social norms (Xiao *et al*., 2022). The idea of translating Bayesian predictive models to study two-person interactions at both the individual and collective levels, based on a dialectical mis-attunement hypothesis in autism, has been proposed (Bolis *et al*., 2017). Such interpersonal paradigms and computational modeling can similarly be used to examine interpersonal deficits in PSZ. However, there are outstanding challenges to understanding social coordination in schizophrenia. For example, it is unclear whether and how knowledge of social coordination in healthy individuals can be applied to PSZ. Additionally, unveiling intrinsic cognitive/neural processes associated with social coordination deficits in schizophrenia remains challenging because these internal processes cannot be directly observed or easily calculated. A possible solution to this problem is to use Bayesian statistics to infer posterior probabilities of inner processes based on actions and interaction outcomes. In conclusion, empirical studies are needed in the future to clarify the cognitive, neural, and computational mechanisms of social coordination deficits in schizophrenia.
